# Clinicopathologic characteristics and treatment outcomes of hepatoid adenocarcinoma of the stomach, a rare but unique subtype of gastric cancer

**DOI:** 10.1186/1471-230X-11-56

**Published:** 2011-05-19

**Authors:** Sun Kyung Baek, Sae-Won Han, Do-Youn Oh, Seock-Ah Im, Tae-You Kim, Yung-Jue Bang

**Affiliations:** 1 Department of Internal Medicine, Seoul National University Hospital, Seoul, Korea; 2 Cancer Research Institute, Seoul National University College of Medicine, Seoul, Korea; 3 Department of Internal Medicine, Kyung Hee University Hospital, Seoul, Korea

**Keywords:** Gastric hepatoid adenocarcinoma, treatment outcome, prognosis, clinicopathologic

## Abstract

**Background:**

Gastric hepatoid adenocarcinoma (HAC) is a special type of gastric cancer that morphologically mimics hepatocellular carcinoma. In this study, we performed an evaluation of clinicopathologic characteristics, treatment outcome, and prognosis in patients with gastric HAC.

**Methods:**

We consecutively enrolled patients with pathologically proven gastric HAC at Seoul National University Hospital between January 1996 and December 2008 and conducted a retrospective review. Among 15,253 patients with gastric cancer, 26 patients (0.17%) were diagnosed as gastric HAC.

**Results:**

Among 26 patients, 22 were male and the median age was 63. Stage at diagnosis was stage IB in 3 patients, stage II in 6 patients, stage III in 7 patients, and stage IV in 10 patients. Eight patients out of 18 patients with stage IB, II, III, and IV relapsed after curative surgery. Relapse-free survival for these patients was 16.67 months. The most common metastatic site was intraabdominal lymph nodes (n = 9), followed by the liver (n = 8). Thirteen patients received palliative chemotherapy. The most commonly used regimen was a combination of fluoropyrimidine and platinum. Partial response was observed in one patient and stable disease in 5 patients. Median overall survival and progression free survival of these patients were 8.03 (95% CI: 6.59-9.47) and 3.47 months (95% CI: 0.65-6.29), respectively.

**Conclusions:**

Gastric HAC is a very rare but unique type of stomach cancer. Early detection of this type of cancer is of critical importance to patient prognosis. Additional studies to reveal the biology of this tumor are warranted.

## Background

Hepatoid adenocarcinoma (HAC) showed a histologic appearance typical of hepatocellular carcinoma, including solid, trabecular, and pseudograndular structures; tumor cells were round or polygonal in shape [1]. HACs have been described in several different organs, including the lung, pancreas, esophagus, ampulla of Vater, colon, urinary bladder, renal pelvis, ovaries, uterus, and cervix [2-12]. The stomach is the organ in which HAC has been most commonly identified. Incidence of HAC is known to be 0.38-0.73%, as reported in a previous study [1,13]

Alpha-fetoprotein (AFP) is a fetal serum protein produced by fetal liver and yolk sac cells, and by some fetal gastrointestinal cells [14]. In adults, serum AFP is elevated in patients with hepatocellular carcinoma, yolk sac tumors, and noncancerous liver disease. AFP-producing tumors have been reported in several different organs [5-7]. The stomach is one of the most common sites affected by these tumors, and the first such case has been described [15]. Later, Ishikura et al. proposed the term "hepatoid adenocarcinoma (HAC) of the stomach" for primary gastric carcinoma characterized histologically by hepatoid differentiation and production of large amounts of AFP [16]. However, he reported observation of primary AFP-negative gastric carcinomas with characteristic histologic features mimicking hepatocellular carcinoma [17]. The clinicopathologic entity was broadened to include gastric carcinoma showing hepatic differentiation without production of AFP. In progression, Nagai et al. noted that diagnosis of HAC of the stomach was not dependent on production of AFP, and suggested that diagnosis should be based on recognition of characteristic histologic features [13].

HACs of the stomach have been known as a rare subtype; therefore, previously reported case reports or case series have focused on aggressiveness or diagnosis, rather than treatment outcome. Information has been very limited. In this study, we conducted an evaluation of clinicopathologic characteristics, treatment outcome, and prognosis in patients with gastric HAC.

## Methods

A computer search of pathology reports was performed for identification of pathologically confirmed hepatoid adenocarcinoma of the stomach from January 1996 to December 2008 in Seoul National University Hospital. Among 15,253 patients with newly diagnosed stomach cancer during this period, 26 patients had hepatoid adenocarcinoma in their pathology reports.

Diagnosis of HAC was dependent on morphologic features. Pathological diagnosis of 23 patients was made primarily by examination of gastrectomy specimens showing histological observations that included specific hepatoid structures, such as the trabecular, pseudo-grandular pattern and hyaline globules; other patients who did not undergo gastrectomy but were strongly suggestive of HAC by biopsy specimens were confirmed by immunohistochemical (IHC) staining (anti-AFP, anti-hepatocyte, alpha-1-antitrypsin) positive and elevated serum AFP level. Incidence of gastric HAC accounts for 0.17% of all gastric cancers.

Pathology reports, medical records, and imagings were reviewed. Clinicopathologic data obtained included age, sex, endoscopic finding, histopathologic characteristics (tumor location, IHC staining, serum AFP levels, history of chronic hepatitis, stage at diagnosis, treatment modality, and overall survival. According to the 7^th ^edition of the American Joint Committee on Cancer (AJCC), stage of tumor was reclassified.

To determine the prognosis of patients with HAC, we assessed relapse free survival (RFS) and overall survival (OS). The starting point of RFS in early or locally advanced malignancy was the first day of surgery, and the starting day of OS was the day of diagnosis. Patients with advanced or relapsed cancer were assessed for overall survival and tumor response to palliative chemotherapy. For evaluation of outcome of palliative chemotherapy, the starting point for overall survival (OS) and progression free survival (PFS) in patients receiving palliative chemotherapy was the first day of chemotherapy. The Kaplan-Meier method was used for estimation of RFS, PFS, and OS. Statistical comparisons and 95% confidence interval were made using an SPSS software package version 13.0.(SPSS Ins., Chicago, IL USA). Tumor response was evaluated according to the Response Evaluation Criteria for Solid Tumors (RECIST).

All patients gave informed consent prior to gastroscopy, surgery, or chemotherapy, and every procedure was performed according to the rules of good clinical practice. This study was approved by the institutional review board of Seoul National University Hospital (IRB No:0911-064-301).

## Results

### 1) Clinicopathologic features of hepatoid adenocarcinoma (HAC) of the stomach

A total of 26 patients were enrolled. Twenty-two patients were male (median age, 63.4 years; range 36.4-74.1 years). Only one patient had chronic hepatitis B. Stage at diagnosis was stage IB in 3 patients, stage II in 6 patients, stage III in 7 patients, and stage IV in 10 patients.

In endoscopic findings, Bormann type 3 was the most common (n = 11), followed by Bormann type 2 (n = 5). Four patients showed the early type of gastric cancer. Tumors occurred mainly at the lower-third, particularly the antrum (n = 16, n = 13, respectively). In IHC, 15 patients were positive for AFP (Dakopatts, Denmark). Only one of four patients with negative AFP was assessed for alpha-1-antitrypsin (Dakopatts, Denmark) and the result was positive. Serum AFP was accessible in 11 patients, of whom 7 patients had elevated levels, and median serum AFP was 208 ng/mL (range 5- 4,750,000 ng/mL) (Table 1).

**Table 1:** Clinicopathologic characteristics of all 26 patients

Characteristics	No of patient	%
Age, median = 63.4 (36.4-74.1)		
30- 60	10	38.5%
> 60	16	61.5%
Sex (n = 26)		
Male	22	84.6%
Female	4	12.5%
Stage at diagnosis (n = 26)		
Stage IB	3	11.5%
Stage II	6	23.1%
Stage III	7	26.9%
Stage IV	10	38.5%
Endoscopic finding (n = 23)		
Borrmann type I	2	8.7%
Borrmann type II	5	21.7%
Borrmann type III	11	47.8%
Borrmann type IV	1	4.3%
EGC^†^	4	17.4%
Location (n = 25)		
Upper-third	2	8.0%
Middle-third	5	20.0%
Lower-third	16	64.0%
Whole stomach	2	8.0%
Immunohitstochemical staining (n = 26)		
AFP (positive/negative/^‡^NA)	15/4/7	
Anti-hepatocyte (positive/negative/^‡^NA)	4/4/18	
Anti-chymotrypsin (positive/negative/^‡^NA)	1/1/24	
Serum AFP (n = 26)		
Elevated (median, range)	7 ( 208 ng/ml, 5-4,750,000 ng/ml)	
Normal	4	
^‡^NA	15	

^† ^Early gastric cancer type, ^‡^Not assessed

Of 23 patients who underwent gastrectomy, 21 patients could be evaluated for findings of endovascular or endolymphatic tumor emboli. Of those, 71% had endovascular tumor emboli and 71% had endolymphatic tumor emboli in surgical specimens (Table 2). Of 8 patients who did not show recurrence, two patients had endovascular tumor emboli and three patients had endolymphatic tumor emboli. Almost all stage IV patients, except for one patient with only vascular tumor emboli, who underwent gastrectomy, had both endovascular and endolymphatic tumor emboli in surgical specimens.

**Table 2:** Pathologic and laboratory findings

Finding	% (No of patient)		
	
	Present	Not present	Not assessed
Endolymphatic tumor emboli (n = 23)	15	6	2
Endovascular tumor emboli (n = 23)	15	6	2

### 2) Treatment pattern and clinical outcomes for all cases of hepatoid adenocarcinoma (HAC) of the stomach

**Figure 1:**
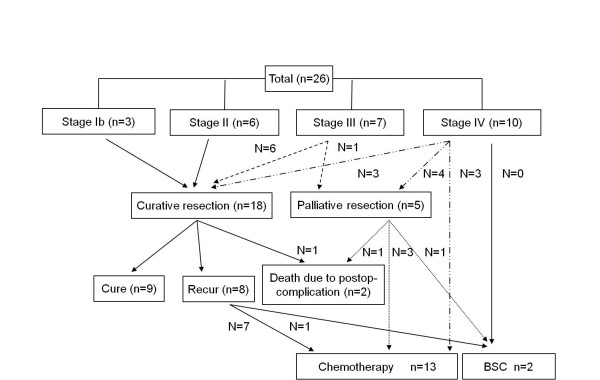
**Treatment patterns for all patients**. Stage at diagnosis was stage IB in 3 patients, stage II in 6 patients, stage III in 7 patients, and stage IV in 10 patients. Nine patients, including three patients with stage Ib, three patients with stage II, one patient with stage III, and 2 patients with stage IV, were cured. Two patients expired due to post-operative complications. Of 10 patients who initially had stage IV, 6 patients underwent gastrectomy and 6 patients received palliative chemotherapy. Two of three stage IV patients who underwent gastrectomy with R0 resection were followed up with no evidence of disease. Seven patients of 16 patients with initial stage I-III relapsed and six patients received palliative chemotherapy.

Figure 1 shows the treatment pattern for all patients. Sixteen patients were diagnosed early or as locally advanced stage and ten patients showed distant metastasis at diagnosis. Of 18 patients undergoing curative resection, 9 patients underwent adjuvant chemotherapy and the other 9 patients did not receive adjuvant chemotherapy due to stage I (n = 3), old age and poor performance (n = 2), patient refusal (n = 2), early relapse (n = 1), and death (n = 1). Two patients with stage II, five patients with stage III, and one patient with stage IV relapsed after curative surgery. Median follow-up duration was 61.4 months (range 4.8-139.5 months). Nine patients, including three patients with stage Ib, three patients with stage II, one patient with stage III, and 2 patients with stage IV, were disease-free. Two patients expired due to post-operative complications (delayed bleeding and sepsis). Of 10 patients who initially had stage IV, 7 patients underwent gastrectomy and 6 patients, including 3 non operable patients, received palliative chemotherapy. Two of three stage IV patients who underwent gastrectomy with R0 resection and metastatectomy were followed up with no evidence of disease, and the other was treated with palliative chemotherapy after recurrence at postoperative 9.2 months. Seven patients of 16 patients with initial stage I-III relapsed and six patients received palliative chemotherapy.

**Figure 2:**
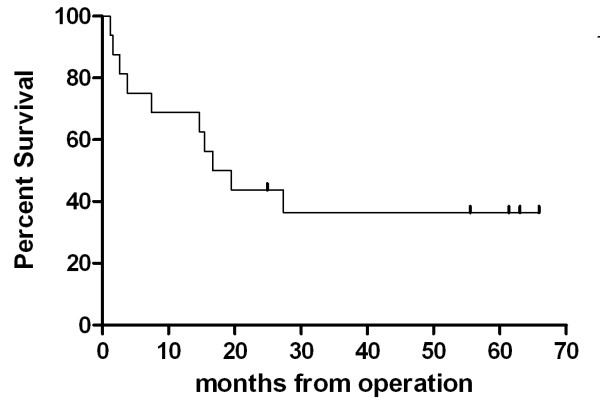
**Kaplan-Meier plot of RFS to early or locally advanced gastric HAC**. Median RFS of patients who underwent curative resection was 16.67 months (range 1.63- 65.9)(Vertical upticks represent alive patients).

**Figure 3:**
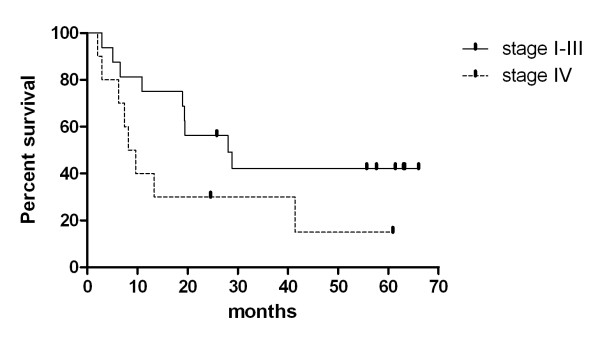
**Kaplan-Meier plot of OS according to stage**. Median OS of stage I-III and stage IV were 28.0 and 8.2 months, respectively (range 2.9 - 66.0 months, 2.1 - 60.87 months, p = 0.068) (Vertical upticks represent alive patients).

Relapse-free median survival of patients who underwent curative resection was 16.67 months (range 1.63- 65.9) (figure 2). Median overall survival of stage I-III and stage IV were 28.0 and 8.2 months, respectively (range 2.9 - 66.0 months, 2.1 - 60.87 months) (figure 3). The survival difference between both group was marginally significant (p = 0.068).

Overall survival of 18 patients with metastatic or recurrent cancer was 9.63 months (95% CI: 4.34-14.92, range: 1.20- 60.87 months).

### 3) Outcomes of palliative chemotherapy in metastatic/recurrent hepatoid adenocarcinoma (HAC) of the stomach

Ten patients had metastatic HAC and 8 had recurrent HAC, of whom 13 patients were treated with palliative chemotherapy. The most common metastatic site was lymph nodes (n = 9), followed by liver (n = 8), and peritoneal seeding (n = 3). Portal vein thrombosis was identified in eight patients.

The most commonly used regimen was a combination of fluoropyrimidine and platinum (n = 7), followed by a combination of paclitaxel and cisplatin, and TS-1 single (respectively n = 2, n = 2)(Table 3). Evaluation of response rate was possible in 12 of 13 patients treated with palliative chemotherapy. One showed partial response and five patients showed stable disease; the response rate was 8.3% and the disease control rate was 50% (95% CI: 25.4 -74.6).

**Table 3:** Clinicopathologic characteristics of patients receiving palliative chemotherapy

Characteristics (n = 13)	No of patient	%
Age,	Median: 59 (36-74)	
Sex		
Male	12	92.3%
Female	1	7.7%
ECOG PS		
0-1	11	84.6
2	2	15.4
Stage		
Stage IV at diagnosis	6	46.2%
Recurred cancer	7	53.8%
Portal vein thrombosis		
Yes	8	61.5%
No	5	38.5%
Metastasis		
LN	9	69.2%
Liver	8	61.5%
Peritoneal seeding	3	23.1%
Pancreas	2	15.4%
lung	1	7.7%
Gastrectomy		
Yes	10	76.9%
No	3	23.1%
Number of metastatic organ		
1-2	8	61.5%
> 3	5	38.5%
Chemotherapy		
Up to 1^st ^line	13	100%
Up to 2^nd ^line	7	53.8%
Up to 3^rd ^line	1	7.7%
Chemotherapeutic agent		
FP/XP/FOLFOX^†^	7	53.8%
TP^‡^	2	15.4%
TS-1	2	15.4%
Others (FL^§^, FM^¶^)	2	15.4%

^†^cisplatin and 5-FU/capecitabine and cisplatin/oxaliplatin, leucovorin and 5-FU

^‡ ^paclitaxel and cisplatin

^§ ^5-FU and leucovorin

^¶ ^5-FU and MMC

**Figure 4:**
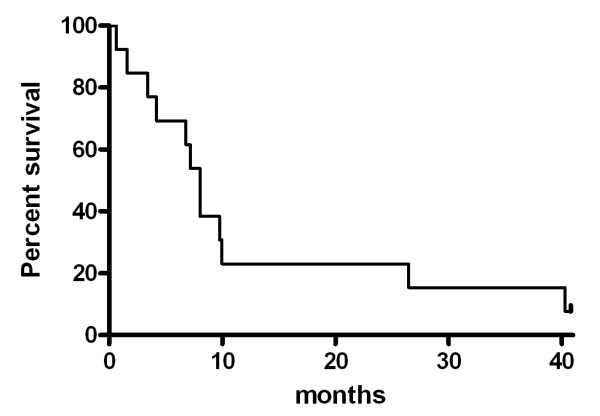
**Kaplan-Meier plot of OS for patients receiving palliative chemotherapy**. Median OS of 13 patients receiving palliative chemotherapy was 8.03 months (95% CI: 6.59-9.47, range: 0.63-40.8 months)

**Figure 5:**
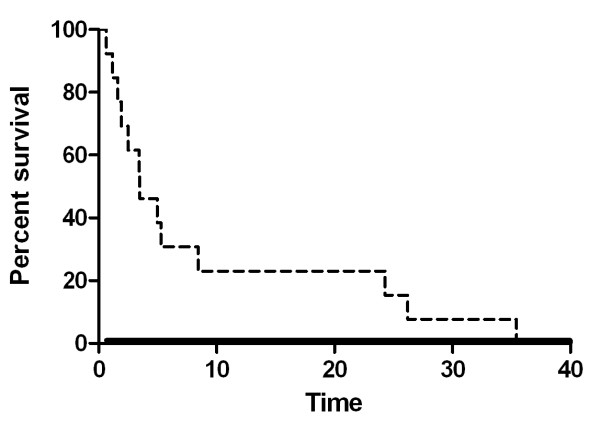
**Kaplan-Meier plot of PFS for patients receiving palliative chemotherapy**. Median PFS of 13 patients receiving palliative chemotherapy was 3.47 months, respectively (95% CI: 0.65-6.29, range: 0.63 - 35.43 months, respectively)

OS and PFS of 13 patients receiving palliative chemotherapy were 8.03 and 3.47 months, respectively (95% CI: 6.59-9.47, range: 0.63-40.8 months, 95% CI: 0.65-6.29, 0.63 - 35.43 months, respectively) (Figures 4 and 5).

## Discussion

In this study, incidence of HAC was 0.17%, which is relatively lower than 0.38-0.73%, which was reported in a previous study [1,13]. Lower incidence of our study is associated with difficulty in diagnosis of this neoplasm. HAC is a rare type of gastric adenocarcinoma and other typical diagnoses should be ruled out. Diagnosis of HAC often required microscopically minute observation and IHC study, including anti-hepatocytes, anti-AFP, and anti-chymotrypsin. Of the total subjects in our study, 88% underwent gastrectomy. Only three patients were diagnosed with HAC without gastrectomy. It is possible that most patients who were diagnosed by biopsy only and who did not require surgery were not included.

Primary gastric cancer could show elevated serum AFP of gastric production of primitive foregut origin, such as the liver, and AFP produced hepatic metastatic lesions; reported incidence of AFP producing gastric cancer has been from 1.3-15% of all cases of gastric cancer. In the early days of HAC definition, it was suggested that HAC developed from AFP-producing gastric cancer in which extensive hepatoid differentiation rarely occurred because not all AFP-producing gastric cancers resemble hepatocellular carcinoma [18]. Later, Ishikura et al. reported on primary AFP-negative gastric carcinomas with characteristic histologic features mimicking hepatocellular carcinoma [17]. Moreover, Nagai et al. reported that 46% of gastric HACs showed negatively stained AFP and that microscopically hepatoid features, regardless of AFP-staining, were more important for prognosis [13]. In their study, all patients with negatively AFP-stained gastric HAC and accessible serum AFP had normal serum AFP levels. Therefore, gastric cancer showing hepatic differentiation without production of AFP was a clinicopathologic entity of HAC.

In gastric HAC, blood vessel and lymphatic permeation and regional lymph node metastasis occurred more frequently than in poorly differentiated gastric adenocarcinoma and primary HCC, and these were associated with a poorer prognosis [1,17]. In our study, of 23 patients who underwent gastrectomy, 71% had endovascular tumor emboli, which was lower than that of a previous study [1]. None of the patients with stage IB had microscopically endovascular or endolymphatic invasion and they did not relapse (median follow up = 61.37 months, range 25.8 - 63.0 months). Six of eight patients who did not relapse did not have endovascular invasion, which may be a good predictor of relapse, although validation will be required. Therefore, diagnosis of gastric HAC at an earlier stage may lead to a better prognosis. The survival difference between stage I-III and stage IV was marginally significant, which might result from small number of patients.

According to a randomized trial for first-line therapy for treatment of advanced gastric cancer, response rate of 1^st ^line chemotherapy is about 32-46% and OS of stage IV stomach cancer is 9.3-10.5 months [19]. In this study, median OS is 8.03 months, which is much longer than that of previous reports that included five patients [20].

Relatively longer survival is associated with good performance and active treatment, including palliative gastrectomy and chemotherapy. In a xenograft model of AFP-producing gastric cancer, 5-FU, doxorubicin, and epirubicin did not induce suppression of tumor growth; however, MMC and cisplatin may be active to some extent [21]. Most patients received platinum based chemotherapy. The disease control rate was 50%. In particular, a patient with initial stage IV and showing partial response to 1^st ^line palliative chemotherapy (cisplatin and 5-FU) showed progression after 26.2 months. The patient also showed partial response to 2^nd ^line cisplatin and paclitaxel.

HAC of the stomach was revised in all of these cases; due to the rare incidence of this neoplasm, the main clinical characteristics of these types of tumors are described in a published report [22]. The majority of patients were male and the average age was about 64 years old. The antrum and pylorus were the most common primary sites of tumors and their AFP levels were much higher than normal. Our data also show male predominance (11:2) and the lower-third was the most common site (65%); 63% of patients checked for serum AFP level had elevated AFP, and 61% of patients were diagnosed as stage I, II, or III, and the other was diagnosed as stage IV. Relapse rate of patients with early stage or locally advanced stage was 47%.

This study also has some limitations. The first is that this is a retrospective study of a single center; thus, we cannot exclude the possibility of a selection bias. The second, sample size is too small. Nevertheless, considering the rare incidence of HAC and previous case reports or case series, this study presents the first report that focused on relapse rate of early or advanced stage HAC and response rate to chemotherapy and treatment outcome of terminal stage HAC.

## Conclusion

In conclusion, HAC of the stomach is a rare type of adenocarcinoma, with an incidence of 0.17% of gastric cancer cases. Of total HAC patients, 60% of patients were diagnosed with early stage or locally advanced stage, and 47% of them relapsed. All patients with early stage gastric cancer were cured. Therefore, early detection of this type of cancer is of critical importance to patient prognosis. Additional studies to reveal the biology and outcome of this tumor are warranted.

## Competing interests

The authors declare that they have no competing interests.

## Authors' contributions

SKB implemented the research hypothesis, coded the data, and drafted the manuscript. DYO conceived of the study and participated in its design and critical revision of the manuscript and approved the manuscript. SWH, DYO, SAI, TYK, and YJB contributed to the patient pool and approved the manuscript. All authors read and approved the final manuscript.

## Pre-publication history

The pre-publication history for this paper can be accessed here:

http://www.biomedcentral.com/1471-230X/11/56/prepub
